# Eicosanoids and cancer

**DOI:** 10.6061/clinics/2018/e530s

**Published:** 2018-08-03

**Authors:** Renata Nascimento Gomes, Souza Felipe da Costa, Alison Colquhoun

**Affiliations:** Departamento de Biologia Celular e do Desenvolvimento, Instituto de Ciencias Biomedicas, Universidade de São Paulo, SP, BR

**Keywords:** Metabolism, Eicosanoids, Prostaglandins, Lipoxygenases, Inflammation

## Abstract

Eicosanoids are 20-carbon bioactive lipids derived from the metabolism of polyunsaturated fatty acids, which can modulate various biological processes including cell proliferation, adhesion and migration, angiogenesis, vascular permeability and inflammatory responses. In recent years, studies have shown the importance of eicosanoids in the control of physiological and pathological processes associated with several diseases, including cancer. The polyunsaturated fatty acid predominantly metabolized to generate 2-series eicosanoids is arachidonic acid, which is the major n-6 polyunsaturated fatty acid found in animal fat and in the occidental diet. The three main pathways responsible for metabolizing arachidonic acid and other polyunsaturated fatty acids to generate eicosanoids are the cyclooxygenase, lipoxygenase and P450 epoxygenase pathways. Inflammation plays a decisive role in various stages of tumor development including initiation, promotion, invasion and metastasis. This review will focus on studies that have investigated the role of prostanoids and lipoxygenase-derived eicosanoids in the development and progression of different tumors, highlighting the findings that may provide insights into how these eicosanoids can influence cell proliferation, cell migration and the inflammatory process. A better understanding of the complex role played by eicosanoids in both tumor cells and the tumor microenvironment may provide new markers for diagnostic and prognostic purposes and identify new therapeutic strategies in cancer treatment.

## BIOSYNTHESIS OF PROSTANOIDS

Prostanoid is a term used to define a family of biologically active lipids containing 20 carbons, which include prostaglandins (PGs) (PGD, PGE and PGF), prostacyclin (PGI) and thromboxane (TXA). These lipids are synthesized from the polyunsaturated fatty acids (PUFAs) dihomo-gamma-linolenic acid (DGLA, precursor of series 1 prostanoids), arachidonic acid (AA, precursor of series 2 prostanoids) and eicosapentaenoic acid (EPA, precursor of series 3 prostanoids). Among these precursors, AA is the most important and predominant in humans 1-4.

PGs were first observed by Kurzrok and Lieb 5 in 1930 in human seminal fluid. This observation was confirmed by von Euler 6 in 1935, and twenty years later, Bergström and Sjövall 7 successfully purified the first PGs, which were subsequently named PGE_1_ and PGF_1α_. In the 1970s, it became clear that PGs have diverse effects on cells, although the mechanisms of action were unknown. It became easier to understand the action of PGs after the identification of their membrane receptors, making this area of research attractive and important 8,9.

Prostanoids are ubiquitous lipids in animal tissues and coordinate a multitude of physiological and pathological processes, either within the cells in which they are formed or in closely adjacent cells in response to specific stimuli. Under normal physiological conditions, prostanoids are involved in the relaxation and contraction of smooth muscles, regulation of blood clotting, maintenance of renal homeostasis, modulation of immune responses, inhibition and stimulation of neurotransmitter release, regulation of gastrointestinal tract secretion and motility and protection of the gastrointestinal mucosa. Prostanoids are also involved in many pathological conditions, such as inflammation, cardiovascular disease and cancer 10-12.

**Figure 1:**
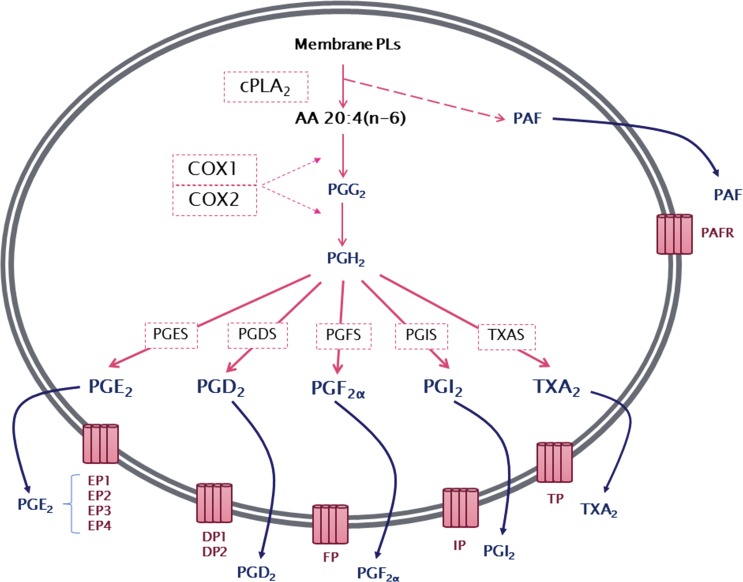
General overview of series-2 prostanoid biosynthesis. After being released from membrane phospholipids (PLs) by the action of cytosolic phospholipase A_2_ (cPLA_2_), arachidonic acid is converted by cyclooxygenase 1 or 2 (COX1 or COX2) to an unstable intermediate, prostaglandin H_2_ (PGH_2_), which is rapidly converted to the PGs PGE_2_, PGD_2_, PGF_2α_, PGI_2_, and thromboxane A_2_ by their specific synthases. Membrane PL cleavage also results in the release of lysophosphatidylcholine, which can be converted to platelet-activating factor (PAF). Prostanoids, thromboxanes and PAF are then released from the cell and can exert a wide range of actions mediated by binding to their specific G protein-coupled receptors, EP1–4, DP1–2, FP, IP, TP and PAFR.

The production of prostanoids occurs through a complex enzymatic pathway (Figure 1). The first step is the activation of cytosolic phospholipase A_2_ (cPLA_2_), which, by hydrolysis, releases AA from membrane glycerophospholipids. PG endoperoxide H synthase 1 or 2, more commonly known as cyclooxygenase 1 or 2 (COX1 or COX2), then catalyzes a reaction in which molecular oxygen is inserted into AA. This reaction produces an unstable intermediate, PGG_2_, which is rapidly converted to PGH_2_ by the peroxidase activity of COX. The resulting PGH_2_ is then modified by specific synthases that generate PGs and TXA, each of which has its own range of biological activities 13-16.

After synthesis, prostanoids can cross the cell membrane by simple diffusion (poorly, due to their charged nature at physiological pH) or can be transported out of the cell by members of the ABC transporter superfamily 17. In the extracellular environment, prostanoids can bind to their specific receptors to activate multiple intracellular pathways 18. There are nine different receptors for the prostanoids: DP1 and DP2 receptors for PGD_2_; EP1, EP2, EP3 (splice variants) and EP4 receptors for PGE_2_; FP receptor for PGF_2α_; IP receptor for PGI_2_; and TP receptor for TXA_2_ 19-21.

The prostanoid receptors can be divided into three groups based on the type of G protein to which they are coupled and consequently the function of the evoked cellular responses. In the first category are the receptors related to relaxant activity, IP, EP2, EP4 and DP, which are usually coupled to Gs (stimulatory) proteins, and their activation stimulates the production of cAMP by adenylate cyclase (AC).

The second category is represented by receptors with constrictor activity, such as EP1, FP and TP, which are coupled to Gq proteins, mediating an increase in intracellular concentrations of Ca^2+^. The third group is represented only by the EP3 receptor, which is coupled to Gi (inhibitory) proteins whose activation inhibits AC, reducing cAMP concentrations. It is important to highlight that despite the specificity of most receptors related to products of the COX pathway, the TP receptor for TXA_2_ can also be stimulated by the prostanoids PGE_2_, PGI_2_ and PGF_2α_ 1,22.

Although most prostanoids bind to cell surface receptors, in some cases, they can bind to nuclear receptors. One of the main targets is the family of peroxisome proliferator-activated receptors (PPARs), which are known to regulate lipid metabolism, cell differentiation and proliferation 23.

The intracellular concentrations of prostanoids are controlled not only by their synthesis but also by their enzymatic degradation. Degradation begins with the transport of prostanoids from the extracellular fluid to the cytoplasm by the PG transport protein (PGT), followed by inactivation by the action of 15-hydroxyprostaglandin dehydrogenase (15-PGDH) 24,25.

This process gives rise to metabolites with very limited biological activities including 13,14-dihydro-15-keto PGF_2α_ for PGF_2α_ and 13,14-dihydro-15-keto PGE_2_ in the case of PGE_2_. PGD_2_ is metabolized to PGs of the J series (PGJ_2_, delta-12-PGJ_2_, 15-deoxy-delta12,14 PGJ_2_) or F series (9α,11β-PGF_2_). TXA_2_ and PGI_2_ are unstable and are rapidly hydrolyzed to their inactive metabolites TXB_2_ and 6-keto- PGF_1α_, respectively 26-28.

## PROSTANOIDS AND CANCER

Many studies over the years have shown the ability of prostanoids to alter cancer cell proliferation and death, influence angiogenesis, increase cell migration and invasion and maintain a state of chronic inflammation 28,29.

Among prostanoids, PGE_2_ is the most abundant PG in the body and is produced by several cells, such as fibroblasts, leukocytes and renal cells. This lipid mediator is the best known member of the PG family, as it plays an important role in several physiological systems, such as the gastrointestinal, renal, cardiovascular and reproductive system, in addition to being the main mediator of inflammation. PGE_2_ is involved in pathological conditions such as cancer 28,30. Elevated concentrations of PGE_2_ are found in several human malignancies, including colon, lung, breast and head and neck cancer, and are often associated with poor prognosis.

The biological relevance of increased production of PGE_2_ in tumors has not yet been fully established. Recently, Brocard et al. 31 demonstrated that the addition of exogenous PGE_2_ to primary glioblastoma (GBM) cultures increased the survival and proliferation of the analyzed cells. In another study, the exogenous addition of PGE_2_ to the T98G human glioma cell line caused a significant increase in cell proliferation and migration, as well as a decrease in apoptosis 32.

The increase in PGE_2_ concentration is often related to the altered expression of COXs, especially COX2. The COX2 enzyme is overexpressed in cancer cells and is associated with progressive tumor growth, as well as the resistance of cancer cells to conventional chemotherapy and radiotherapy. Evidence shows that increased COX2 expression and subsequently increased downstream PGE_2_ release contribute to the repopulation of tumors and consequent inefficient treatment 30,31.

In the work of Murakami et al. 33, increased expression of COX2 and mPGES1 in the HEK-293 cell line increased cell proliferation. In addition, increased expression of COX2 and mPGES1 in the same cells injected into the flanks of nude mice was responsible for the formation of large, well-vascularized tumors. Treatment of HCA-7 colon carcinoma cells with the mPGES1 inhibitor CAY10526 decreased PGE_2_ production and attenuated cell proliferation, while increasing mPGES1 expression, PGE_2_ production and cell proliferation 34.

In the case of COX1, Osman and Youssef 35 observed a high expression of COX1 in 62.5% of renal cancer tissues. As renal carcinoma tumor grade progressed from grade I-IV, COX1 expression progressively increased in comparison with that in normal renal tissues.

In the work of Cheng et al. 36, PGE_2_ promoted increased migration of Huh-7 hepatocarcinoma cells through its EP2 receptor. In the PC3 prostate cancer cell line, the migration induced by PGE_2_ was mediated, in part, by EP4 37. In the CCLP1 and HuCCT1 liver cancer cell lines, the increase in migration caused by the addition of PGE_2_ occurred through the EP3 receptor 38.

Currently, proteins involved in the transport and degradation pathways of PGE_2_ are gaining increasing attention, since the procarcinogenic effects of PGE_2_ are regulated not only by their biosynthesis but also by their degradation. The internalization and inactivation of PGE_2_ are performed by two distinct proteins. PGE_2_ is transported into the cells through PGT and subsequently oxidized to 15-keto-PGE_2_ by 15-PGDH. Both steps are necessary for the efficient inactivation of PGE_2_ 39. Studies have shown that PGT and 15-PGDH expression is often reduced in several neoplasms 26,40.

The analysis of 15-PGDH expression by qRT-PCR and western blotting revealed low expression in the breast cancer cell lines MCF-7, T-47D, BT-474, ZR75-1, MDA-MB-231, MDA-SK-BR-3 and BT-20 25. In high-grade neuroblastoma, low expression of 15-PGDH and consequent high concentrations of PGE_2_ were identified relative to those in low-grade neuroblastomas 41. These studies suggest that changes in PGE_2_ levels may play a crucial role in tumor development.

TXA_2_ plays a central role in homeostasis and is increasingly implicated in cancer progression. TXA_2_ production has been shown to be increased in human mammary carcinomas relative to that in normal breast tissues and was related to increased tumor size and metastatic potential, as well as the absence of estrogen (ER) and progesterone receptors (PR) 42. Additionally, the TXA_2_ receptor TP in triple negative breast cancer (TNBC) enhanced cell migration and invasion and activated Rho signaling, phenotypes that could be reversed using Rho-associated kinase (ROCK) inhibitors. TP also protected TNBC cells from DNA damage by negatively regulating reactive oxygen species (ROS) levels 43. In prostate cancer, activation of TP led to cytoskeletal reorganization and rapid cell contraction through the activation of the small GTPase RhoA, while blockade of TP activation compromised tumor cell motility 44,45.

In another study, analysis of TP mRNA levels in 120 human breast tumors and 32 noncancerous mammary tissues showed that higher levels of TP transcripts were significantly associated with higher grade tumors and shorter disease-free survival 46. High expression levels of TP have also been observed in lung, bladder and prostate cancer cell lines, leading to increased cell proliferation, migration and invasion capacity 44,45,47. In lung cancer cells, thromboxane A_2_ synthase (TXAS) inhibited apoptosis via negative regulation of ROS production in the lung 48. The use of furegrelate, a potent inhibitor of TXAS, in addition to inhibiting the migration process, decreased adhesion, increased apoptosis, decreased tumor growth *in vivo* and increased sensitivity to radiation in glioma-derived cells 49-52.

In the literature, PGF_2α_ is related to increased migration and invasion observed in colorectal carcinoma cells 53. In prostate cancer, the overexpression of aldo-keto reductase 1C3 (AKR1C3), an enzyme involved in PG metabolism, resulted in the accumulation of PGF_2α_, which not only promotes prostate cancer cell proliferation but also enhances prostate cancer cell resistance to radiation 54.

Keightley et al. 55 observed that in endometrial cancer, PGF_2α_ induced activation of the FP receptor in the epithelial cells of endometrial adenocarcinoma, resulting in the stimulation of the calmodulin-NFAT signaling pathway. This signaling pathway leads to elevated ADAMTS1, which functions in an autocrine/paracrine manner to promote epithelial cell invasion and in a paracrine manner to inhibit endothelial cell proliferation 55.

In endometrial cancer, the binding of PGF_2α_ to the FP receptor enhanced cell proliferation, migration and angiogenesis of carcinoma cells through the activation of the extracellular signal-regulated kinase (ERK) pathway 56. Müller et al. 57 also found downregulation of the FP receptor in skin papillomas in mouse models of cancer, and its level of expression was inversely correlated with PGF_2α_ production, suggesting that PGF_2α_ regulates levels of the FP receptor in the squamous epithelium. Scott et al. 58 also demonstrated that in melanoma, the concentrations of PGF_2α_ were higher than those in normal melanocytes. These results show that the binding of PGF_2α_ to the FP receptor activates signals that stimulate a differentiated phenotype. In addition, PGF_2α_ concentration was found to be consistently higher in breast cancer than in benign and non-neoplastic tissues 59,60.

Evidence from the literature suggests that PGI_2_ may protect against cancer development by inhibiting tumor growth, angiogenesis, invasion and metastasis, and thus can be considered a potential chemopreventive agent. Studies have indicated that the lungs of mice treated with PGI_2_ had 40–50 times fewer metastatic nodes than lungs of mice treated with the positive control . The same study showed that treatment of mice with PGI_2_ resulted in a 10% decrease in the adhesion of metastatic cells to endothelial tubules 61.

Preclinical chemoprevention studies showed that pulmonary PGI synthase (PGIS) overexpression and elevated PGI_2_ concentrations protect against lung tumorigenesis in a variety of murine tumorigenesis models, including those established by exposure to tobacco smoke 62,63. The administration of tranylcypromine, which inhibits PGIS, has been shown to reduce cancer multiplicity in murine carcinogenesis models, indicating that the inhibition of this enzyme may be useful in the chemoprevention of breast cancer 64.

In primary human lung tumor samples, loss of PGIS mRNA was observed relative to that in matched normal controls 65. These findings are in agreement with the study by Stearman et al. 66, in which gene expression analysis of non-small cell lung cancer (NSCLC) showed a loss of PGIS in human lung tumor samples. However, a small group of adenocarcinoma patients whose lung tumors retained PGIS expression were found to have significantly enhanced survival. A statistically significant correlation was also observed in head and neck squamous cell carcinoma. Patients who expressed high levels of PGIS in head and neck squamous cell carcinoma tissues had a higher 5-year survival rate than patients with low levels of PGIS 67.

In the case of breast cancer, analysis revealed that the expression of PGIS is associated with a reduction in patient survival 68. On the other hand, the expression of the prostacyclin receptor IP appears to indicate an angiogenic phenotype of tumor endothelial cells according to a study in which migration and tube formation were inhibited by the IP receptor antagonist RO1138452 69. These apparently contradictory actions of PGI_2_ on cell survival may indicate that its effects are highly dependent on the specific cellular environment.

The implications of PGD_2_ production in tumor development and progression have remained largely unexplored. The few studies on this prostanoid consider PGD_2_ to have an antitumor activity 70. This hypothesis is supported by studies showing that elevated levels of PGD_2_ result in relatively few metastatic foci in rat lungs, inhibition of leukemic cell growth and Ehrlich tumor growth, and decreased metastatic potential in melanomas 71-73.

In a study by Park et al. 74, which evaluated the possible influence of PGD_2_ on the development of intestinal adenomas, a 50% increase in intestinal adenomas was shown in the ApcMin/knockout mouse model for the hematopoietic PGD synthase (H-PGDS) enzyme, whereas in the ApcMin/+ mouse model with high expression of H-PGDS, an approximately 80% reduction in the adenomas was observed.

In gliomas, lower protein and mRNA levels of lipocalin-PGD synthase (L-PGDS), the main PGDS produced in neurons and glial cells, were observed in different GBM samples than in normal brain tissues 75. Moreover, the exogenous addition of PGD_2_ to the A172 and C6 lines resulted in a decrease in the proliferative capacity of the cells 75,76. Recent studies have confirmed that PGD_2_ can inhibit glioma cell proliferation. However, at lower physiological concentrations of PGD_2_, U87MG, U251MG and A172 glioma cell proliferation and migration were stimulated rather than inhibited 77

The correlation between hepatic metastasis and PGD_2_ concentration in human cancer tissues has also been studied. The mean PGD_2_ concentration in primary cancer tissues was significantly lower in the group with hepatic metastasis than in the group without hepatic metastasis 78. Using gastric cancer cells, Fukuoka et al. 79 observed that PGD_2_ significantly decreased the proliferation of tumor cells via the PPARγ pathway. In 277 human gastric tumors, L-PGDS-positive cases were significantly correlated with PPARγ-positive cases. In recent years, several studies have shown that PGD_2_ has antiproliferative activities and can induce cellular apoptosis via the activation of caspase-dependent pathways in human leukemia cells 80,81 and colon cancer cells 78.

In a study with A549 lung carcinoma cells, PGD_2_ induced cell death through the intrinsic apoptotic pathway, and similar results were also found with another lung carcinoma cell line, H2199. Moreover, the generation of 15-deoxy-delta12,14 PGJ_2_, a metabolite of PGD_2_, seems to be the key factor responsible for the apoptosis observed in A549 cells 82.

## LIPOXYGENASE PATHWAY

### 5-Lipoxygenase (5-LOX)

**Figure 2:**
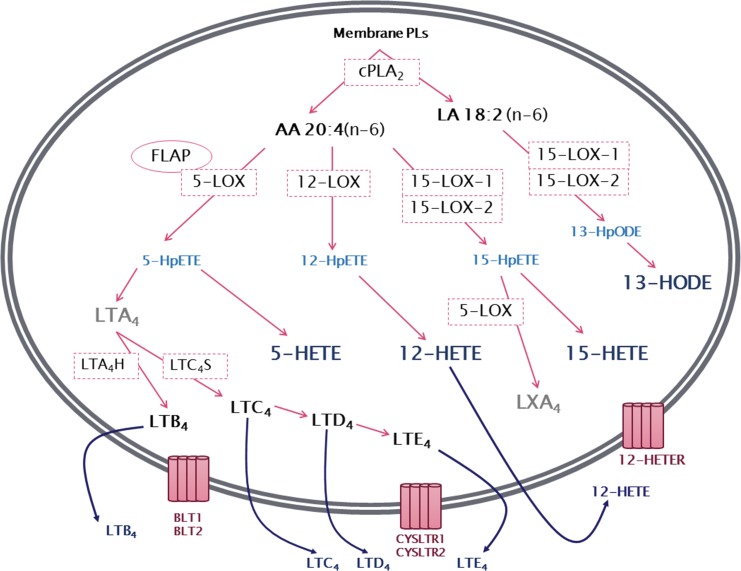
General overview of leukotriene biosynthesis. After being released from membrane phospholipids (PLs) by the action of cytosolic phospholipase A_2_ (cPLA_2_), arachidonic acid is converted by the lipoxygenases (LOXs) 5-LOX, 12-LOX and 15-LOX-1 or 15-LOX-2 to the corresponding hydroperoxyeicosatetraenoic acid (HpETE) - 5-HpETE, 12-HpETE or 15-HpETE. These are rapidly converted to hydroxyeicosatetraenoic acids (HETEs) - 5-HETE, 12-HETE and 15-HETE. In addition, 5-HpETE is catalyzed by 5-LOX to form the unstable leukotriene LTA_4_, which, through the action of LTA_4_ hydrolase, results in the synthesis of LTB_4_. Alternatively, LTA_4_ can be converted into the cysteinyl leukotriene LTC_4_ by the action of LTC_4_ synthase. LTC_4_ can then be converted to LTD_4_ and LTE_4_. Linoleic acid (LA) can be metabolized by 15-LOX producing 13-hydroperoxyoctadecadienoic acid (13-HpODE), which is then metabolized to 13-hydroxyoctadecadienoic acid (13-HODE). 15-HpETE can also be catalyzed by 5-LOX, often transcellularly, to produce lipoxin A_4_ (LXA_4_). Leukotrienes and HETEs are then released from the cell and can exert a wide range of actions mediated by binding to their specific receptors, BLT1-BLT2, CysLTR1-CysLTR2 and 12-HETER.

The 5-lipoxygenase (5-LOX) pathway is the most well characterized among the lipoxygenase pathways. It begins with the insertion of molecular oxygen and the formation of a hydroperoxyl group at carbon 5 of the AA chain, resulting in 5-hydroperoxyeicosatetraenoic acid (5-HpETE) that can be converted to 5-hydroxyeicosatetraenoic acid (5-HETE) 83. 5-HpETE can also be converted to leukotriene A_4_ (LTA_4_), a leukotriene with no known biological activity that serves as a precursor for the synthesis of biologically active leukotrienes. The conversion of LTA_4_ by the enzyme LTA_4_ hydrolase (LTA_4_H) results in the production of leukotriene B_4_ (LTB_4_), while the enzyme LTC_4_ synthase produces leukotriene C_4_ (LTC_4_) (Figure 2). LTC_4_, in turn, is the precursor of the other members of the cysteinyl leukotrienes including LTD_4_ and LTE_4_. 12,84. Leukotrienes and 5-HETE have similar effects on neutrophils and other leukocytes, serving as potent chemoattractants in addition to modulating adhesion, migration and degranulation. However, little is known about the specific receptors of 5-HETE 85. Several studies indicate that 5-HETE is a substrate for 5-hydroxyeicosanoid dehydrogenase (5-HEDH), resulting in the synthesis of the non-classical eicosanoid 5-oxo-eicosatetraenoic acid (5-oxoETE), with a much more potent effect than 5-HETE on neutrophils 86,87.

Lepley et al. 88 were the first to show the mechanisms controlling the activation of 5-LOX, identifying the presence of phosphorylated 5-LOX in activated neutrophils, thereby correlating the activity with its phosphorylation. The regulation of the enzymatic activity of 5-LOX depends on its interaction with Ca^2+^, interaction with the FLAP protein and 5-LOX translocation through cellular compartments. Additionally, 5-LOX is phosphorylated at residues Ser271, Ser663 and Ser523 by the activity of MAPKAP2 (Ser271), ERK2 (Ser663) and protein kinase A (PKA) (Ser523). Several additional phosphorylation sites, at Tyr42, Tyr53 and either Tyr94 or Tyr445, were recently identified by Markoutsa et al. 89. Once bound to Ca^2+^, 5-LOX translocates to the nuclear envelope. The FLAP membrane protein, with affinity for AA, is found in the nuclear envelope. The exact mechanisms that regulate the relationship between FLAP and 5-LOX are still not fully understood, but once 5-LOX is activated and is present in the nuclear envelope, FLAP acts as a substrate carrier that presents AA to the 5-LOX enzyme 90-93.

The expression of 5-LOX is usually low or absent in normal tissues but detected in response to pathological conditions in cells derived from bone marrow, such as granulocytes, macrophages, and B lymphocytes 94. The leukotrienes and 5-HETE generated by 5-LOX play an important role in the inflammatory process associated with numerous diseases including cancer, allergic asthma, dermatitis, rhinitis, arthritis, atherosclerosis, ischemia and septic shock 95,96.

The relationship between 5-LOX and cancer has been explored in the literature over the past two decades. In the late 1990s, an *in vitro* study with PC-3 and LNCaP prostate cancer cell lines showed that MK886, a FLAP-binding 5-LOX inhibitor, leads to apoptosis due to the inhibition of 5-LOX activities. Furthermore, the addition of 5-HETE or 5-oxo-ETE was sufficient to prevent the effects of MK886 97.

Later studies identified 5-LOX as a potential biomarker for malignancy. Larré et al. 98 found higher concentrations of LTB_4_ in prostate carcinoma tissues than in peritumoral tissues. Another study with 42 patients analyzed 5-LOX expression by immunohistochemistry in brain tumor samples. With the exception of three low-grade gliomas, all samples showed 5-LOX expression 99. Additionally, 5-LOX expression in 111 colon adenomas showed a correlation with high risk factors that traditionally are markers for malignant transformation to colorectal adenocarcinoma, thereby providing clues about the link between 5-LOX and colorectal cancer malignancy 100. Leukotriene receptors were also found to be altered in cancer, with increased BLT1 expression in prostate 101 and colon carcinoma 102, and the pharmacological blocking of BLT1 activity was sufficient to reduce cell proliferation.

The data accumulated so far, notably in prostate and colorectal carcinoma, points to an important role of 5-LOX during tumor development and progression. Recently, the physiological role of 5-LOX products as chemoattractants and stimulators of myeloid cells was correlated with their role in cancer progression 103. Interestingly, evidence shows that 5-LOX activity in mast cells is important for the promotion of abnormal cell proliferation during intestinal polyposis in mice 104. In another recent study, the importance of microenvironment-derived 5-LOX products was assessed by injecting Lewis lung carcinoma cells into 5-LOX-deficient rats to compare tumor growth with that in control rats 105. The tumor microenvironment of 5-LOX-deficient rats showed an increase in angiogenesis and reduction in neutrophils and cytotoxic T cells, leading to larger tumors than in control rats. Although the importance of 5-LOX in tumor development and progression is convincing, the roles played by 5-LOX metabolism from the tumor microenvironment *versus* the tumor cells are still not fully elucidated.

### 12-Lipoxygenase (12-LOX)

12-Lipoxygenase (12-LOX) is another enzyme in the lipoxygenase family, responsible for the insertion of molecular oxygen and the formation of a hydroperoxyl group at carbon 12 of the AA chain to form 12-HpETE. Similar to 5-HpETE, 12-HpETE has no known biological activity, serving as a precursor for 12-HETE production (Figure 2).

The former name 12-LOX platelet-type was due to the detection of 12-HETE in platelets during the 1970s, and 12-HETE was subsequently characterized as an endothelial retraction factor 106. 12-LOX is expressed in smooth muscle cells, keratinocytes, endothelial cells, macrophages and platelets, and the physiological roles of 12-HETE are associated with lymphatic vessel permeability and smooth muscle cell retraction to modulate vessel contraction 107. Furthermore, 12-HETE displays both antithrombotic and prothrombotic activities through the modulation of platelet aggregation 108-110.

One of the earliest correlations between 12-LOX and cancer was reported in a study with 112 samples from radical prostatectomy, where an increase in 12-LOX expression was found to correlate with advanced stage, poor differentiation and invasive potential according to pathological stage, histological grade and surgical status 111. Since then, many studies have found that 12-HETE is strongly correlated with metastasis. Similar to the activities of other eicosanoids, 12-HETE activities are triggered through its recognition by specific receptors on the plasma membrane. A specific G protein-coupled receptor, GPR31, also called 12-HETE receptor (12-HETER), was recently identified 112. The activation of 12-HETER leads to protein kinase C (PKC) activation, stimulating the PKC/ERK1/2 pathway and altering cell proliferation 113. Furthermore, 12-HETE release downregulates E-cadherin and stimulates endothelial cell migration, increasing lymphatic vessel permeability 114. Additionally, a similar mechanism is responsible for increased endothelial barrier permeability by 12-HETE 115. By altering vascular permeability, 12-HETE plays a key role in neutrophil migration through the endothelial barrier and also modulates RhoA-dependent migration (116, 117).

By acting on vascular permeability, cell attachment and cell migration, 12-HETE can facilitate tumor cell migration through the endothelial barrier, thereby facilitating metastasis. Chen et al. 118 showed a correlation between 12-HETE production and metastasis by treating C57BL/6J mice with the selective 12-LOX inhibitor N-benzyl-N-hydroxy-5-phenylpentamidine (BHPP). A reduction in lung colonies was observed in animals treated with BHPP. Another study showed *in vitro* that the same inhibitor could attenuate endothelial cell migration and proliferation in response to angiogenic factors. In addition, 12-LOX inhibition significantly reduced angiogenesis *in vivo*. 119. In the MKN-28 gastric cancer cell line, inhibition of 12-LOX with baicalein induced apoptosis 120. Not only the inhibition but also the upregulation of 12-LOX in colorectal cancer cells led to changes in proliferation and migration. The induced overexpression of 12-LOX in these cells increased migration and metastasis in mice 121. Reinforcing the role of 12-LOX in metastasis, the secretion of 12-HETE by MCF-7 breast cancer cell spheroids co-cultured over a lymphatic endothelial monolayer induced circular discontinuities due to endothelial retraction 122. As previously mentioned, these effects on endothelial cells have been proposed to be an important step in 12-HETE-stimulated metastasis. More recently, in MCF-7 or MDA-MB-231 breast cancer cell spheroids co-cultured with lymphatic endothelial cells, 12-HETE was shown to increase intracellular Ca^2+^ release in endothelial cells, inducing a Ca^2+^-dependent disruption in their barrier functions and increasing the number of discontinuities in the endothelial monolayer 114,123.

### 15-Lipoxygenases (15-LOX-1 and 15-LOX-2)

Human 15-lipoxygenases (15-LOXs) are a subfamily formed by two isoforms: 15-LOX-1 and 15-LOX-2. 15-LOX-1 (also known as 15-LOX reticulocyte-type) was initially identified in rabbit reticulocytes and is normally expressed in eosinophils, reticulocytes and respiratory epithelia 124. Both 15-LOX-1 and 15-LOX-2 were classified and named based on their ability to insert molecular oxygen and form a hydroperoxyl group at carbon 15 of AA, producing 15-HpETE, which can be reduced to 15-HETE (Figure 2). Although 15-LOX-1 and 15-LOX-2 can both use AA as a substrate, both molecules can also oxygenate the 18-carbon fatty acid linoleic acid (LA) at carbon 13 to produce 13-hydroperoxyoctadecadienoic acid (13-HpODE) that can then be reduced to 13-hydroxyoctadecadienoic acid (13-HODE) 125. However, the substrate specificity of the two enzymes is not identical. The 15-LOX-1 enzyme has a higher affinity for LA (therefore producing 13-HODE), while 15-LOX-2 shows a preference for AA (therefore producing 15-HETE) and poorly metabolizes LA 126,127.

Due to differences between their activities and differences in substrates/products, both 15-LOX-1 and 15-LOX-1-2 are correlated with many pathological processes associated with chronic inflammation, such as in asthma, atherosclerosis, insulin resistance and cancer 128-130. The preference of 15-LOX-1 to metabolize LA to 13-HODE has been correlated in many studies with a protective effect in inflammatory diseases. Depletion of 15-LOX-1 is proposed to exacerbate inflammation in atherosclerosis, encephalomyelitis, asthma and osteoarthritis 131-134. The roles of 15-HETE, on the other hand, are associated with inflammatory processes and angiogenesis. Zhang et al. 135 demonstrated that 15-HETE stimulation led to the migration and formation of endothelial cell tubes *in vitro* in a process dependent on PI3K-AKT-mTOR activation. Wang et al. 136 showed the functional role of 15-HETE and the 15-LOX pathway in angiogenesis in a mouse stroke model, consistent with the literature in which an increase in 15-HETE concentrations followed post-ischemic hypoxia in neural tissues 137-139.

Regarding the roles of 15-LOX-1 and 15-LOX-2 in cancer, one of the first studies to propose an antitumorigenic role for 15-LOX-1 showed a decrease in 13-HODE production and 15-LOX-1 expression in 18 colon cancer samples compared with those in normal colon samples 140. Several subsequent studies have provided support for the antitumorigenic role of 13-HODE in specific tumors. Shureiqi et al. 141 showed in colorectal cancer cell lines that treatment with the specific COX2 inhibitor celecoxib caused apoptosis following an increase in 13-HODE concentration. Interfering with 13-HODE production by silencing 15-LOX-1 protected celecoxib-treated cells from death. Another study using HCT-116 and HT-29 colon cancer cells, which do not have detectable levels of 15-LOX-1, showed that the induced expression of 15-LOX-1 significantly decreased cell proliferation and increased apoptosis. Furthermore, a decrease in adhesion to fibronectin, anchorage-independent growth on soft agar, and migratory and invasive capacity on Matrigel was observed to strongly associate 15-LOX-1 activity with the inhibition of migration and metastatic capacity in colon cancer 142. This association between 15-LOX-1 and an antitumorigenic role was also reported in a study with weaker 15-LOX-1 in 120 breast cancer tissue samples than in normal tissues, indicating that the loss of 15-LOX-1 expression may be correlated with tumorigenesis 143. Additionally, in pancreatic cancer cell lines and pancreatic carcinoma samples (n=12), 15-LOX-1 expression was found to be reduced. Stable expression 15-LOX-1 in pancreatic cancer cell lines was found to reduce proliferation 144. Exogenous treatment of 13-HODE in breast and colorectal cancer cells also caused a reduction in cell viability 141,145.

Although a number of studies suggest the antitumorigenic role of 13-HODE, others show a different role for 15-LOX-1 and 13-HODE in the prostate. The prostate cancer cell lines PC-3 and LNCaP have high concentrations of 13-HODE. Moreover, treatment of PC-3 cells with 13-HODE led to enhanced MAP kinase (MAPK) pathway signaling, resulting in increased proliferation 146. Kelavkar et al. 147 observed strong 15-LOX-1 expression in 48 prostatectomy samples with varying degrees of malignancy, and the expression level of 15-LOX-1 was positively correlated with p53 mutations and the degree of malignancy. Sen et al. 148 evaluated the adenovirus-mediated overexpression of 15-LOX-1 by injecting adenoviruses harboring 15-LOX-1 with green fluorescent protein (GFP) or GFP alone into the dorsolateral prostates of C57BL/6 mice. After 90 days, the expression of 15-LOX-1 resulted in the development of a prostate intraepithelial neoplasia-like phenotype, increasing the expression of Ki-67 as well as the angiogenic markers FGF-a and FGF-b. The study thus proved that the forced overexpression of 15-LOX-1 in normal prostate tissue is enough to increase cell proliferation and upregulate genes associated with malignancy.

While 13-HODE and 15-LOX-1 are generally believed to play an antitumorigenic role, data on 15-LOX-2 are less conclusive. Most research data available on 15-HETE refers to the 12/15-LOX rodent isoform, which can metabolize AA to form both 12-HETE and 15-HETE, making it more difficult to correlate the data with human 15-LOX-2 149. Despite previous *in vitro* results reporting the overexpression of 15-LOX-2 in breast cancer cell lines 150, in breast tumor biopsy samples (n=120), 15-LOX-2 expression was decreased in comparison with that in normal tissues 143. In lung cancer, a recent study treated NSCLC cell lines with both 13-HODE and 15-HETE and found a decrease in proliferation, induction of apoptosis and activation of peroxisome proliferator-activated receptor γ (PPARγ) 151. While 15-LOX-1 is overexpressed and 13-HODE is present at higher concentrations in prostate cancer, 15-LOX-2 and 15-HETE are reportedly decreased in high-grade prostate neoplasia 152, suggesting opposing roles for 15-LOX-1 and 15-LOX-2 in prostate cancer. The work of Hsi et al. 146 corroborated these opposite effects of 15-LOXs by showing that in the PC-3 prostate cell line, 13-HODE upregulated the activity of MAPK and increased the phosphorylation of PPARγ, while 15-HETE downregulated MAPK and decreased PPARγ phosphorylation.

The role of eicosanoids and their degradation products in the development and progression of cancer has been a target of investigations for many years. Despite considerable study, many controversies still exist in the literature in relation to individual eicosanoids in specific tumor settings. As we have highlighted in this review, many eicosanoids are considered to be tumorigenic, some are believed to be antitumorigenic, and several have mixed properties dependent on the tumor type in question. Clearly, the complex interplay among the eicosanoid pathways, their products, their receptors and the subsequent intracellular signaling pathways that are activated need to be better delineated and remain important subjects for future studies. An important goal in these studies will be to provide a better understanding of the complex role played by eicosanoids in both tumor cells and the tumor microenvironment. Such detailed information could provide new diagnostic and/or prognostic markers and identify new therapeutic strategies in cancer treatment.

## AUTHOR CONTRIBUTIONS

Gomes RN, Souza FC and Colqunhon A contributed to the literature review and writing of the manuscript.
